# Enhancing knowledge, attitude, and perceptions towards fall prevention among older adults: a pharmacist-led intervention in a primary healthcare clinic, Gemas, Malaysia

**DOI:** 10.1186/s12877-024-04930-5

**Published:** 2024-04-02

**Authors:** Priya Manirajan, Palanisamy Sivanandy, Pravinkumar Vishwanath Ingle

**Affiliations:** Department of Pharmacy Practice, School of Pharmacy, International Medical University, 57000 Kuala Lumpur, Malaysia

**Keywords:** Older adults, Aging, Multimorbidity, Medications, FRIDS, Awareness

## Abstract

**Background:**

Falls and fall-related injuries are very common among older adults, and the risk of falls increases with the aging process. The lack of awareness of falls and fall-related injuries among older adults can contribute to an increasing risk of falls. Hence, a study was carried out to improve the knowledge, attitude, and perception of falls and fractures among older adults in a primary care setting in Gemas, a rural area of the Selangor state of Malaysia.

**Method:**

A structured educational intervention was provided to older adults who visited the primary care setting in Gemas and provided written informed consent to participate in the study. A total of 310 older adult patients was included in the study using a convenience sampling technique.

**Results:**

Before the intervention, 74.84% of the respondents (*n* = 232) agreed that falls and related fractures are the leading causes of hospital admission among older adults. In post-intervention, the number of respondents who agreed with this statement increased to 257 (82.91%). At baseline, 28 respondents (9.03%) had poor knowledge, 160 respondents (51.61%) had average knowledge levels, and 122 respondents (39.35%) had good knowledge. In post-intervention, respondents with poor and average knowledge reduced to 1.93% (*n* = 6) and 29.35% (*n* = 91) respectively. A majority of respondents’ knowledge levels improved significantly after the intervention (*n* = 213; 68.71%). About eight respondents (2.58%) had a negative perception of falls. In post-intervention, the percentage reduced to 0.65% as only two respondents had a negative perception. A total of 32 types of fall-risk-increasing drugs (FRIDs) have been prescribed to the respondents. A strong correlation (*r* = 0.89) between pre- and post-intervention knowledge was shown among the respondents. Paired t-test analysis showed a statistically significant difference.

**Conclusion:**

The pharmacist-led educational intervention significantly improved the knowledge, attitude, and perception of falls among older adults. More structured and periodical intervention programmes are warranted to reduce the risk of falls and fractures among older adults.

**Supplementary Information:**

The online version contains supplementary material available at 10.1186/s12877-024-04930-5.

## Introduction

Fall incidents among older adults have a negative impact on their personal lives and the economy as well. It burdens the health care sector as well because of the treatment cost. A past history of a fall is a significant predictor of future fall risk [1–3]. Hence, it is essential to find solutions to prevent fall incidents, and fall-related injuries and fractures. As falls contribute to increased mortality, reduction of quality of life, increased hospitalization, and medical costs, this has led to the development of prevention strategies [4–6]. Multifactorial prevention measures have been identified and implemented in various countries. Many interventional studies have been conducted in the past that show prescribing multiple medications is likely to increase the risk of falls. Several drugs can increase the risk of falls and are termed fall-risk-increasing drugs (FRIDs) which include antihypertensives, antihistamines, sedatives-hypnotics, antipsychotics, antidepressants, opioids, and non-steroidal anti-inflammatory drugs [6–8]. Around one-third of older adults experience at least one fall incident every year while 10% of them experience multiple falls [1]. An earlier study found that amlodipine, a FRID increases the risk of falls among older adults compared to chlorthalidone (HR: 2.24; *p* = 0.03) or lisinopril (HR: 2.61; *p* = 0.04) [6]. Polypharmacy and FRIDs were the most common cause of falls reported by many studies [6–8], 74.19% (*n* = 230) of older adults were prescribed polypharmacy (Mean: 5.18 ± 0.64), and 22.85% (*n* = 69) received four types of medications, majorly cardiovascular medications, that might have increased the risk falls in their population [6].

Deprescribing FRIDs may benefit in preventing falls among older adults [7, 8]. Besides deprescribing FRIDs and assessment of knowledge, attitude, and perception (KAP), an educational intervention was found to help reduce fall incidents among older adults [9–11]. Optimistic fall outcomes such as reducing the risk of falls can be achieved by the utilisation of multiple intervention strategies [7]. These strategies include medication reviews, home safety checklists, fall brochures, and many more [12]. Educational materials are useful as they provide information and tips on ways to avoid falls. Any health programme’s outcome will be more effective if older adults take a more active role in fall prevention strategies.

Compared to other developed countries such as North America and Europe, fall prevention strategies and programmes in Southeast Asia are still in their infancy, as the aging population in these countries is expected to surpass in the future [13–15].. The majority of studies conducted to date in Malaysia only assess the prevalence of falls as well as identify the risk factors that contribute to falls [16–18]. Furthermore, these studies did not include older adult patients at primary care clinics; instead, they focused on older adults in hospital or community settings. Nonetheless, a pharmacist-led fall intervention study is scarce in the country. A multidisciplinary approach including educational intervention by pharmacists has proven to be effective in controlling chronic medical conditions and better health outcomes among the patient population. Pharmacists doesn’t only dispenses medications, but they do play a vital role in the health care sector to improve the health of the patient population. Educational intervention by pharmacists is essential, especially for older adults with fall risk to be aware of their medical condition and medications [19–21]. Hence, health care policies governing fall prevention measures in primary care settings have yet to be developed. To fulfill the future demands arising from the increasingly aging population, changes in the current health policies are required. Thus, this will be the first study of its kind conducted in Malaysia as well as in Asia assessing the KAP of falls among older adults in primary healthcare, reviewing FRIDs and offering FRIDs intervention, and providing a pharmacist-led educational intervention to improve the outcome.

## Methods

### Study design, population, and sample recruitment

An interventional study was carried out in a primary care setting in Gemas, Malaysia. The data collection targeting older adults was carried out from February to August 2022. The participants who were 65 years and above and seeking medical treatment at this primary clinic were included in the study. The participants must be able to read, understand, and respond to the questionnaire and study materials provided to them. Participants with hearing and visual impairment were not included in the study. Participants who met the inclusion criteria and were willing to participate in the study were recruited by the clinical pharmacist who is one of the researchers in the current study. The older adults seeking medical treatment at this study site were approached by the clinical pharmacist, who provided an explanation of the study’s purposes and requested them to participate in the present study.

The study was approved by the institutional research and ethics committee, the head of the facility of the study setting. Confidentiality of all the information gathered during the data collection process was maintained throughout the study. Informed written consent was obtained from each participant prior to enrolling in the study.

### Study questionnaires and intervention materials

The validated study questionnaire was obtained from a published study by Gamage et al. which investigated knowledge and perception of falls among older adults in Sri Lanka [22]. Prior permission was obtained to use the questionnaire. The study questionnaire was modified and validated with an additional set of questionnaires on attitudes towards falls. The study questionnaire was sectioned into two parts that examined the participants’ demographic characteristics, and KAP of falls. The knowledge component consists of eight subcomponents assessing risk factors associated with falls and fractures, sites of fall-related fractures, fall prevention, and sources of information related to falls and fractures. The attitude and perception component, on the other hand, consists of eight statements each, targeting falls and falls-related fractures. A total of 26 questions were used in this study. The initial questionnaire was designed in English and later was translated and validated in Bahasa Melayu as most participants only comprehend the national language. The study questionnaire is presented in Supplementary Table 1.

### Intervention procedure

**Fig. 1:**
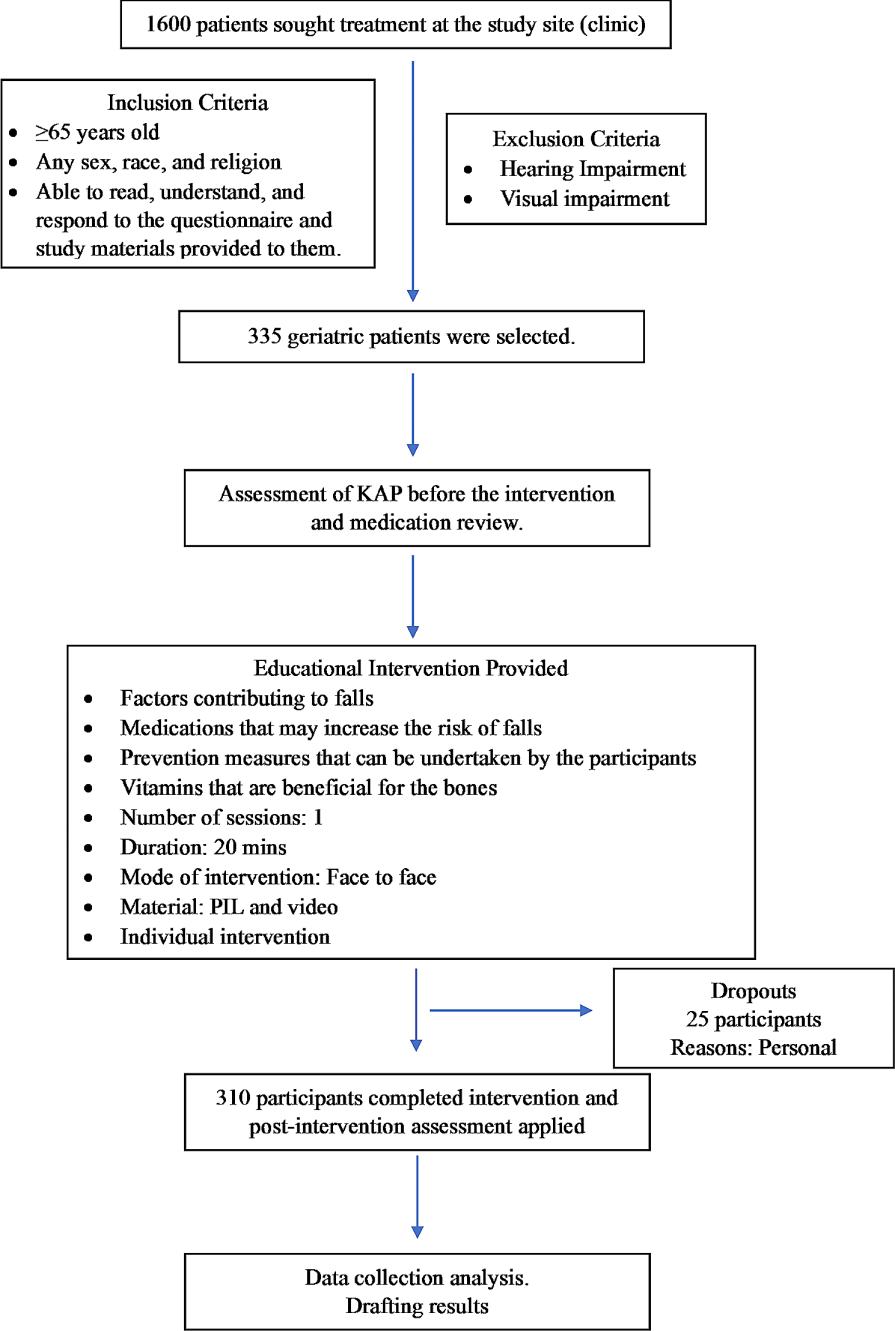
A schematic diagram of the study design and the intervention procedures

As the study was conducted among older adults, all the participants were included in the intervention group, and no control group was created. The KAP of falls before the intervention was assessed for every individual participant by the clinical pharmacist. The questionnaires were disseminated to the participants while waiting to be examined by the doctors. Once the KAP of falls had been assessed, a fall prevention poster and video (educational intervention materials) were made available to all the participants in the study. The educational intervention on falls, fall risk, complications, and FRIDs was provided individually through one-to-one pharmacist-patient interview sessions. After the educational intervention was provided, KAP was assessed again on the same day. Pre- and post-KAP assessments were carried out by face-to-face interviews. While intervention material was being disseminated, a medication review for each participant was carried out to identify potential FRID. Intervention on the FRID medications was done after consulting the prescriber regarding the potential increased risk of falls when consuming FRID medications. A duration of 15–20 min was spent on the intervention process for each participant. The details are shown in Fig. 1 schematic diagram.

### Statistical analysis

The sample size for the present study was calculated using Raosoft® sample size calculator, with a 5% margin of error, 95% confidence interval, and 50% response distribution. The calculated sample size for this study was 310. Statistical Package for Social Sciences (SPSS) version 26.0 was used to tabulate and analyse the data. The correlation between pre- and post-intervention was assessed using Pearson’s correlation test. The paired t-test was used to determine the mean difference between pre- and post-intervention. A *p*-value of less than 0.05 was considered statistically significant.

Based on the previous study conducted by Gamage et al. [22]. each positive response to knowledge-based questions was given a mark of five. A mark of zero was given to each negative response. The maximum mark an individual could be obtained was 100. The total score was divided into three categories. An individual scored between 0 and 33 was considered to have poor knowledge, scores between 34 and 66 were categorised as average knowledge, while individuals with scores of 67 to 100 were deemed to have good knowledge.

Each statement under the perception and attitude section was scored based on a Likert scale ranging from 1 to 5. The maximum number of marks that could be obtained was 40, with a minimum of 08. Individuals with scores of 8–24 were considered to have negative perceptions or attitudes, while those with scores of 25–40 were considered to have positive perceptions or attitudes [22].

## Results

### Respondent’s demographic characteristics

In the present study, the majority of the respondents are females (*n* = 169; 54.52%), followed by males (*n* = 141; 45.48%). A majority of the respondents fall between the age group of 65 and 69 (*n* = 171; 55.15%), followed by 70 to 74 years (*n* = 104; 33.57%), and the mean age was 69.72 (SD: 2.85) years and about 74% of the participants obtained primary-level education. Less than half of the respondents (*n* = 120; 38.71%) experienced falls after the age of 65 years, and 63 respondents (20.32%) experienced one or more incidents of falls within the last 12-month period. The demographic details are presented in Table 1.

**Table 1:** Demographic data and characteristics of the respondents (*n* = 310)

Socio-demographic Characteristics	Frequency (%) (*n* = 310)
**Sex**	
Female	169 (55)
Male	141 (45)
**Age (years)**	
65–69	171 (55.15)
70–74	104 (33.57)
75–79	29 (9.35)
80–84	6 (1.93)
≥ 85	0
**Ethnicity**	
Malay	308 (99)
Indian	2 (1)
**Educational Status**	
No formal education	12 (4)
Primary education	229 (74)
Secondary Education	69 (22)
**Marital Status**	
Married	217 (70)
Widowed	94 (30)
Do you satisfy with the facilities you have to access health care services?	
Yes	306 (99)
No	4 (1)
Previous experience of fall incidents among the respondents	
Yes	120 (39)
No	190 (61)
Incidents of falls among the respondents within the last 12 months period	
Yes	63 (20)
No	247 (80)
Allergies	
Yes	296 (95)
No	14 (5)

### Respondent’s comorbidities and fall -risk- increasing drugs (FRIDs)

A majority of the respondents in this study were diagnosed with multiple comorbidities (*n* = 295; 95.16%). Most of them (*n* = 139; 44.84%) were diagnosed with three types of diseases which were type 2 diabetes mellitus (T2DM), hypertension (HTN), and dyslipidaemia. Twenty of them (6.45%) had been diagnosed with four types of diseases, and 15 respondents (4.84%) were only diagnosed with a single medical condition which is either HTN, dyslipidaemia, chronic obstructive pulmonary disease (COPD), or myocardial infarction (MI). The details are presented in Supplementary Table 2.

A total of 32 types of FRIDs has been prescribed to the respondents. A majority of the FRIDs belong to the class of cardiovascular medications (*n* = 23; 71.88%), followed by endocrine (*n* = 5; 15.62%), and central nervous system (CNS) (*n* = 4; 12.50%). The majority of the respondents (*n* = 259; 83.55%) were prescribed simvastatin, amlodipine (*n* = 232; 74.84%), perindopril (*n* = 163; 52.58%), and metformin (*n* = 171; 55.16%). The details are presented in Supplementary Table 3.

.

**Table 2:** Respondent’s knowledge level on fall (*n* = 310)

Statements	Pre-intervention (*n* = 310)		Post-intervention (*n* = 310)		*p*-value
	Yes n (%)	No n (%)	Yes n (%)	No n (%)	
**According to your knowledge, which of the following are risk factors for falls among people in your age?**					
Biological factors such as age, gender, visual impairment, chronic diseases	241 (77.74)	69 (22.26)	296 (95.48)	14 (4.52)	0.00*
Unsafe environment	237 (76.45)	73 (23.55)	303 (97.74)	7 (2.26)	0.00*
Behavioural factors such as lack of physical activity, alcoholism	144 (46.45)	166 (53.55)	241 (77.74)	69 (22.26)	0.00*
Socioeconomic factors (low income, difficulties to accessing health facilities)	74 (23.87)	236 (76.13)	134 (43.23)	176 (56.77)	0.00*
Medication/Medicines	82 (26.45)	228 (73.55)	216 (69.68)	94 (30.32)	0.00*
None of the above	38 (12.26)	0 (0)	1 (0.32)	0 (0)	0.00*
**Medical conditions that may lead to a person falling include**,					
Parkinson’s disease	10 (3.23)	300 (96.77)	37 (11.94)	273 (88.06)	0.00*
Hypertension	236 (76.13)	74 (23.87)	263 (84.84)	47 (15.16)	0.00*
Diabetes	81 (26.13)	229 (73.87)	116 (37.42)	194 (62.58)	0.00*
Bone disorders	63 (20.32)	247 (79.68)	100 (32.26)	210 (67.74)	0.00*
None of the above	55 (17.74)	0 (0)	25 (8.06)	0 (0)	0.00*
**A fall may result in**,					
Reduced mobility	280 (90.32)	30 (9.68)	293 (96.52)	17 (5.48)	0.00*
Restriction of activities	281 (90.65)	29 (9.35)	288 (92.90)	22 (7.10)	0.01*
Social isolation	139 (44.84)	171 (55.16)	138 (44.52)	172 (55.48)	0.32
None of the above	24 (7.74)	0 (0)	13 (4.19)	0 (0)	

*p-value < 0.05 considered as statistically significant

### Respondent’s knowledge of falls, risk factors, and fall prevention

Before the intervention, the majority of the respondents (*n* = 232; 74.84%) agreed that falls and related fractures are the leading causes of hospital admission among older adults while 31 respondents (10.0%) disagreed, and 47 (15.16%) said don’t know. In post-intervention, the number of respondents who agreed with this statement increased to 257 (82.91%) while the number of respondents who disagreed and were unsurely decreased to 25 (8.06%) and 28 (9.03%), respectively. A strong correlation (*r* = 0.89) between pre- and post-intervention knowledge was shown among the respondents. Paired t-test analysis showed a statistically significant difference.

For the question “According to your knowledge, which of the following are risk factors for falls among people in your age?” the majority of the respondents during pre-intervention answered that the biological factors (*n* = 241; 77.74%) and unsafe environment (*n* = 237; 76.45%) can be risk factors for falls. In post-intervention, the number of respondents who agreed to both the risk factors increased to 296 (95.48%) and 303 (97.74%) respectively. The improvement after the intervention showed a significant positive correlation with r-values of 0.37 and 0.27 respectively. The majority of the respondents (*n* = 166; 53.55%) during pre-intervention didn’t agree that behavioural factors can be a risk for falls, however, in post-intervention, 241 respondents (77.74%) agreed that it can be a risk factor for falls (*r* = 0.49). The majority of the respondents (*n* = 228; 73.55%) didn’t agree medications can contribute to falls among older adults, during pre-intervention. In post-intervention, more than half (*n* = 216; 69.98%) of the respondents agreed that medications can be a risk factor for falls (*r* = 0.39). Paired t-test analysis showed statistically significant differences for each question.

Prior to intervention, about 55 (17.74%) respondents didn’t agree with any of the suggested medical conditions that may lead to falling. Post-intervention showed improvement in the knowledge whereby the number of respondents agreed that the following medical conditions that may lead to a person falling; Parkinson’s disease (*n* = 37; 11.94%; *r* = 0.44), hypertension (*n* = 263; 84.84%; r-value = 0.73), diabetes (*n* = 116; 37.42%; *r* = 0.75), and bone disorders (*n* = 100; 32.26%; r-value = 0.73). Paired t-test analysis showed statistically significant differences for each question. The details are presented in Table 2. The details of respondents’ knowledge of fall prevention and fall risk factors are presented in Supplementary Tables 4 and 5.

Before the intervention, most of the respondents agreed that falls may result in reduced mobility (*n* = 280; 90.32%) and restriction in daily activities (*n* = 281; 90.65%). Some respondents felt that social isolation (*n* = 139; 44.84%) can be experienced by someone who fell. Around 24 respondents (7.74%) were unsure of the outcome of falls. In Post-intervention, the number of respondents who agreed that falls can cause reduced mobility and restriction of activities increased to 293 (96.52%; *r* = 0.74; *p* = < 0.05) and 288 (92.90%; *r* = 0.86; *p* = = 0.01). However, the number of respondents who agreed to social isolation decreased by one (*n* = 138; 44.52%; *r* = 0.99).

The majority of the respondents felt that proper nutrition (*n* = 283; 91.29%), regular exercise and active lifestyle (*n* = 281; 90.65%), proper medication intake (*n* = 294; 94.84%), and a conducive environment such as good lighting, clean and clutter-free floor (*n* = 289; 93.23%) can prevent older adults from falling. In post-intervention, their knowledge was significantly improved. The details are presented in Supplementary Table 4.

### Respondents’ attitude and perception of falls and fractures

The respondents’ perception of falls and fractures was analysed during pre-and post-intervention. During the pre-intervention, for the first statement, few respondents strongly agreed (*n* = 50; 16.13%) and agreed (*n* = 70; 22.58%) that there is nothing that can be done to prevent falls. In post-intervention, the majority of the participants disagreed (*n* = 208; 67.10%) with the statement. During the pre-intervention, 107 respondents (34.52%) disagreed that they were weak and needed to do fall intervention activities. In post-intervention, the majority of the respondents agreed (*n* = 157; 50.65%) that they are weak and need to do fall intervention activities. In post-intervention, the majority of the respondents (*n* = 219; 70.65%) agreed that the intervention given after the first fall can prevent recurrent falls. Similarly, about one-third of the respondents (*n* = 100; 32.26%) were unsure that carrying out a knowledge training programme in fall-induced injuries in the community is a great necessity. After the intervention, majority of the respondents (*n* = 227; 73.22%) agreed that a knowledge training programme could be beneficial for the community. A positive correlation between pre- and post-intervention was obtained. The details are presented in Supplementary Tables 6 and 7.

### Intervention on FRIDs medications

The intervention was provided to all the participants, and their prescriptions were reviewed for the appropriateness of prescribed medications. The prescriptions were reviewed for medication appropriateness, dose, frequency, duration, possible side effects, and inclusion of FRIDs. Upon the pharmacist’s medication review, there were 15 prescriptions found to have FRIDs with a potential chance of causing fall-related injuries. FRIDs prescribed in these 15 prescriptions were amended by the prescribers upon pharmacist recommendations by replacing the drugs with suitable alternatives (*n* = 5; 33.33%), deprescribing (*n* = 3; 20.0%), and dose alteration (*n* = 7; 46.67%). The details are presented in Table 3.

**Table 3:** The outcome of FRIDs medications review (*n* = 310)

The outcome of the medication review	Frequency (%)	Pharmacist recommendation
Change of therapy	5 (1.61)	a) Change of basal-bolus insulin to premixed insulin (*n* = 1) b) Change amlodipine to perindopril (*n* = 1) c) Change bolus insulin to gliclazide (*n* = 1) d) Change simvastatin to atorvastatin (*n* = 2)
Dose alteration	3 (0.97)	a) Reduce dose and frequency of metformin 500 mg BD to 750 mg OD (*n* = 1) b) Reduce the dose of metformin from 1 g BD to 500 mg BD (*n* = 1) c) Reduce dose of premixed insulin from 14iu BD to 10iu BD (*n* = 1)
Deprescribing	7 (2.26)	a) Off prazosin & perindopril (*n* = 1) b) Off gliclazide & amlodipine (*n* = 1) c) Off perindopril (*n* = 2) d) Off HCTZ (*n* = 1) e) Off frusemide (*n* = 1) f) Off bisoprolol & HCTZ (*n* = 1)
No change in therapy	295 (95.16)	-

### Effectiveness of educational intervention

The effectiveness of the educational intervention was assessed based on the scores assigned to each appropriate answer. At baseline, 28 respondents (9.03%) had poor knowledge, 160 respondents (51.61%) had average knowledge levels, and 122 respondents (39.35%) had good knowledge. In post-intervention, respondents with poor and average knowledge reduced to 1.93% (*n* = 6) and 29.35% (*n* = 91) respectively. A majority of respondents’ knowledge levels improved significantly after intervention (*n* = 213; 68.71%). About eight respondents (2.58%) had a negative perception of falls. In post-intervention, the percentage reduced to 0.65% as only two respondents had a negative perception. A similar scenario was observed in attitudes towards falls and fractures where the negative attitude was observed in seven respondents (2.26%) prior to intervention and after the intervention, only two respondents were observed with a negative attitude. The differences in post-intervention scores were found to be statistically significant. The results are presented in Table 4.

**Table 4:** Effectiveness of educational intervention on respondent’s KAP (*n* = 310)

Domain	Pre-intervention		Post-intervention		*p*-value
	N (%)	Mean score ± SD	N (%)	Mean score ± SD	
Knowledge Score					
0–33 (Poor knowledge)	28 (9.03)	60.05 ± 14.434	6 (1.93)	72.58 ± 12.661	0.00*
34–66 (Average knowledge)	160 (51.61)		91 (29.35)		
67–100 (Good knowledge)	122 (39.35)		213 (68.71)		
Perception Score					
08–24 (Negative perception)	8 (2.58)	29.16 ± 1.749	2 (0.65)	29.82 ± 1.746	0.00*
25–40 (Positive perception)	302 (97.42)		308 (99.35)		
Attitude Score					
08–24 (Negative attitude)	7 (2.26)	28.03 ± 1.004	2 (0.65)	28.72 ± 1.173	0.00*
25–40 (Positive attitude)	303 (97.74)		308 (99.35)		

*p-value < 0.05 considered as statistically significant

The effectiveness of the educational intervention was compared between respondents’ educational level as well as sex. Improvements in KAP scores were seen after educational intervention provided to respondents with no formal education (*n* = 12; 3.87%), primary education (*n* = 229; 73.87%), and secondary education (*n* = 69; 22.26%). The details are presented in the Supplementary Table 8.

## Discussion

The current study evaluates the effectiveness of pharmacist-led educational intervention in improving the KAP of falls, fractures, and FRIDs among older adults and revealed that in post-intervention the majority of the current study participants had a good level of knowledge, a positive perception, and attitude towards falls. The medication review revealed that 32 types of FRIDs had been identified and 4.8% of respondents intervened for FRIDs. Generally, older adults are known to have multiple comorbidities. Multiple co-morbidities are more common with aging causing visual impairment, muscle weakness, a decline in organ function, and so forth. Long-standing medical conditions such as hypertension and diabetes in addition to the aging process will worsen the health condition [23–25]. The comorbidities identified in this study reflect the overall prevalence data collected from the Malaysian National Health and Morbidity Survey (NHMS) conducted in 2019, pointing out T2DM, hypertension, and dyslipidaemia being the major non-communicable disease in this country [26]. As this study was carried out at a primary care clinic in a rural area, complex morbidities such as dementia and osteoporosis were not detected. Patients with high morbidity burden tend to obtain specialist services at secondary or tertiary health care facilities commonly situated in the urban area [27–29]. The maximum number of comorbidities a respondent has detected in this study is up to four.

In the present study, 32 types of FRIDs have been identified. The majority of these belong to the cardiovascular medication classes. A majority of the respondents were taking prescription medication of four or more. Polypharmacy may indicate the presence of multiple FRIDs being prescribed to a single patient which in turn increases the risk of falls. Aging combined with multiple FRIDs are strong indicators of falls. There is evidence showing that multiple FRIDs either from the same class of medications or combining different classes such as cardiovascular and psychotropic medications can lead to increased fall incidents [30, 31]. There were considerably less psychotropic agents detected in this study probably because in this locality not many residents with psychiatric disorders or are seeking medical care at a tertiary care facility. There are numerous studies published have focused on the impact of psychotropic agents and falls [32, 33]. Cardiovascular medications such as diuretics, antihypertensive agents, and statins greatly affect the risk of falls. Statins use may exacerbate a decline in muscle mass leading to reduced muscle performance thus increasing the risk of falls among older adults [34–36]. Besides muscle weakness, statins are also found to affect balance and gait performances in older adults [37].

Prior to educational intervention, a majority of the respondents were aware that biological factors and unsafe environments are risk factors for falls. This awareness comes from their own experience either from falling or observing other older people who experience falls. Initially, a majority of the respondents disagreed that behavioural issues such as lack of physical activity and alcoholism can be risk factors for falls. This can be due to a lack of awareness and low health literacy. Their understanding was improved after providing educational intervention. The socioeconomic status of older adults refers to an individual’s social standing, which is typically measured by several indicators such as occupation, income, education, and wealth [38]. Hence, older adults with poor socioeconomic status have an increased risk of falls as compared to older people with higher socioeconomic status [38, 39]. In Malaysia, the majority of the citizens obtain health care services from public health facilities as the services provided are heavily subsidised by the government. Due to this reason, the majority of the respondents don’t feel the socioeconomic status is a risk factor for falls. Besides easily accessible health care services, lack of awareness that it can be a risk factor could also be a possible reason. A similar situation was encountered when respondents asked if medications can be a risk factor for falls. The majority of them didn’t agree so because they feel medications bring benefits rather than harms. In the educational material provided, an explanation was given on how some classes of medications can contribute to the risk of falling through side effects. As compared to a study conducted in Sri Lanka, respondents from the current study had average knowledge at baseline [22]. In post-intervention, there was a significant improvement in respondents’ knowledge regarding medications as a possible risk factor for falls.

In the present study, respondents do perceive that fall is a preventable event after educating them about fall prevention measures. This group of respondents was aware that they were susceptible to falling. The respondents also perceive that the safety of the house is adequate and educational intervention does not have much impact on this perception. This can be probably due to the majority of fall incidents being experienced in outdoor settings. Less than a majority, perceive weakness negatively thus denying that they require help to prevent them from falling. Educational interventions help to change their negative perception to positive ones as they accept that they are frail and require fall prevention activities [40–42]. The majority of the respondents were aware that recurrent falls can be prevented, if necessary, and steps are taken to avoid falling. They also agree that knowledge training programmes on fall-related injuries should be implemented in the community as they are beneficial. Currently, knowledge training programmes on fall-related injury are not widely implemented, particularly in the rural areas of Malaysia. Despite the education level and locality, the respondents do understand the importance of taking care of their health.

A majority of the participants don’t adjust their beds as they still use conventional bed frames. Hence, the educational intervention does not show any effect on this attitude. Attitude in terms of stopping medications when experiencing side effects improved after the intervention. Educating older adults about the possible side effects of medications and subsequent countermeasures is vital to avoid falls. This activity can be carried out during medication review and counselling by pharmacists. This is a novel study in the Malaysian setting analysing the attitude of older adults towards falls. So forth, no such studies have been carried out in the Malaysian setting, and hence this study will provide an insight into the attitude of older adults towards their experiences and practices towards falls. Moreover, the findings of this study may help healthcare providers in the management of fall risk and will help them to improve the quality of life of older adults. The outcomes reported in the current study are consistent with outcomes reported from other Asian studies proving that educational intervention does improve one’s attitude and perception towards fall [22, 43].

Out of 310 prescriptions reviewed, only 4.8% of prescriptions were found to have FRIDs with a potential chance of inducing falls and were intervened. Pharmacotherapies were not changed for the remaining respondents as the treatment was found to be more beneficial than harmful. Based on a prospective cohort study done by Van Der Velde et al. FRIDs intervention was effective in reducing fall incidents with an absolute risk reduction of 19% and relative risk reduction of 49%. Most of the intervention in the present study was done for cardiovascular medications as a majority was diagnosed with cardiovascular diseases. Since a majority of the intervention was done on cardiovascular medications, the risk reduction of fall incidents might be similar to the above-mentioned study.

Based on the statistical analysis carried out, the pharmacist-led educational intervention was found to be significantly effective in improving the respondent’s KAP of falls. This is evident by the scores reported before and after the intervention had been provided. The result from the statistical analysis also pointed out that the educational materials used in this study benefit the patients regardless of sex and education level. The educational materials can be used even for patients without formal education living in the rural areas.

Pharmacist-led education intervention was proven to be effective in controlling chronic medical condition(s). An earlier study conducted among the Malaysian diabetic population showed that pharmacist-led education intervention significantly improved their knowledge of diabetes and medication adherence behaviour [44]. Another study reported that Pharmacist-led educational interventions have reduced the rate of medication errors [45], and improved health outcomes, particularly medication adherence among patients with hypertension [46]. A study among asthmatic patients revealed that pharmacist-led educational intervention has shown a positive impact on the knowledge of asthma and self-management of the disease [47].

As the study was conducted only in a rural area of a single state, the findings may not represent the actual knowledge, attitude, perception, and practice of older adults in the whole country. Moreover, the ethnicity distribution in this study does not represent the nation’s ethnicity distribution as 99% of the respondents who participated in the study are Malays. There is a possibility of recall bias as some of the respondents may need to recall past events such as fall incidents.

The study was carried out in a rural area with a limited number of respondents, and hence it is recommended to extend the study to other parts of the country with a greater number of older adults to improve the generalisability. It is also recommended to carry out the interventional study with a control group and also for a longer duration of time with follow-up to assess the reduction in fall risk with educational intervention provided to older adults.

## Conclusion

The findings of the study concluded that the older adults in this primary care setting had good knowledge, attitude, and perception (KAP) of falls and fractures in post-intervention. The review of the fall risk-increasing drugs (FRIDs) among older adults found that most of the patients were receiving polypharmacy or multiple medications that significantly contributed to the development of falls. The educational intervention provided to the respondents was significantly effective in improving the awareness of falls and FRIDs. More robust and comprehensive educational interventions on the falls and FRIDs are needed to prevent the occurrence of falls among the wider older adult population.

### Electronic supplementary material

Below is the link to the electronic supplementary material.

Supplementary Material 1

Supplementary Material 2

## Data Availability

The datasets used and/or analysed during the current study are available from the corresponding author on reasonable request.
