# Supplementary material for: Enhancing knowledge, attitude, and perceptions towards fall prevention among older adults: a pharmacist-led intervention in a primary healthcare clinic, Gemas, Malaysia
Source: BMC Geriatr. 2024 Apr 2;24:309. doi: 10.1186/s12877-024-04930-5 (PMC10988811; doi:10.1186/s12877-024-04930-5)
Supplement: Supplementary file 1 — Supplementary Material 1 [file 12877_2024_4930_MOESM1_ESM.docx]

**Supplementary Table 1** Study Questionnaire

Enhancing knowledge, attitude, and perceptions towards fall prevention among older adults: A pharmacist-led intervention in a primary healthcare clinic, Gemas, Malaysia

Serial No: Date of interview:

# Section A: Socio - Demographic information.

1. Date of birth:­­­­­­­___________ (Day/Month/Year) / Age (in years):

2. Race/Ethnicity: Malay / Chinese / Indian / Others (Please state):___________

3. Gender: Male Female

4. Marital Status: Married Single Widowed Divorced

5. Education Level

| No formal education |  | Degree |  |
| --- | --- | --- | --- |
| Primary Education |  | Master’s |  |
| Secondary Education |  | PhD |  |
| Diploma/Certificate |  |  |  |

| <1000 |  |
| --- | --- |
| 1000 - 2000 |  |
| 2000 - 5000 |  |
| >5000 |  |

6. Employment Status: 7. Monthly Income (RM):

| Own Business |  |
| --- | --- |
| Government |  |
| Private |  |
| Retired |  |
| Unemployed |  |

8. Living with: Husband/Wife Children Husband/Wife and Children Alone

9. Did you get any assistance from anyone in the house for your daily chores? Yes No

10. Are you satisfied with the facilities available to access health care services? Yes No

11. Did you ever experience any fall after the age of 65 years? Yes No

12. (a) Within the last 12 months period, did you experience any falls? Yes No

(b) If “Yes”, how many falls did you face within the last 12 months periods?

One Two More than 2 times

(c) If you have a history of falls of more than 1 within the past year, what was the time duration between the first fall and second fall?

≥6 months 7 – 12 months

13. Past medical condition(s)

| Name of the condition | Duration | Medication | Duration of this medication |
| --- | --- | --- | --- |
|  |  |  |  |
|  |  |  |  |
|  |  |  |  |
|  |  |  |  |
|  |  |  |  |

14. Present medical condition(s)

| Name of the condition | Duration | Medication | Duration of this medication |
| --- | --- | --- | --- |
|  |  |  |  |
|  |  |  |  |
|  |  |  |  |
|  |  |  |  |
|  |  |  |  |

15. Details of FRIDs

| Name of the FRID | Frequency | Duration | Indication | Side effects (if any) |
| --- | --- | --- | --- | --- |
|  |  |  |  |  |
|  |  |  |  |  |
|  |  |  |  |  |
|  |  |  |  |  |
|  |  |  |  |  |

16. Do you have any allergies? Yes No

**Section B: Knowledge of Falls and Fractures**

**17.**  Falls and related fractures are the leading causes of hospital admission among elderly people

Yes No Don’t know

18. According to your knowledge, which of the following are risk factors for falls among people your age? (You can choose more than one answer)

- 1. Biological factors such as age, gender, visual impairment, chronic diseases
  2. Unsafe environment
  3. Behavioral factors such as lack of physical activity, alcoholism
  4. Socioeconomic factors (low income, difficulties to accessing health facilities)
  5. Medication/Medicines
  6. None of the above
  7. Others: _________________________ (Please specify)

19. Medical conditions that may lead to a person falling include,

1. Parkinson’s disease
2. Hypertension
3. Diabetes
4. Bone disorders
5. None of above
6. Others: ________________ (Please Specify)

20. What are the common sites of fall related fractures among elderly people?

_____________________________________________

21. A fall may result in,

- 1. Reduced mobility
  2. Restriction of activities
  3. Social isolation
  4. None of above
  5. Others: ­­­­­­­­­­­­­­­­­­­______________________ (Please Specify)

22. Falls prevention knowledge

| Description | Yes | No | Don’t know |
| --- | --- | --- | --- |
| (a) Proper nutrition is very important to maintain your bone and muscle health which helps to protect you from falls. |  |  |  |
| (b) Regular exercise and an active lifestyle help to reduce the chance of falling |  |  |  |
| (c) When taking medication, if you follow proper medical advice, you can minimize the chances of falls due to side effects of drugs. |  |  |  |
| (d) Under favorable circumstances with good lighting, clean and clutter-free floor, the risk of falls can be lowered. |  |  |  |

1. Name two nutrients that are helpful for healthy bones.

……………………………………………………………………..

1. What are the sources of information that you had got above knowledge on falls and fractures?

(You can select more than one answer)

| Radio |  |  | Television |  |  | Newspapers |  |
| --- | --- | --- | --- | --- | --- | --- | --- |
| Doctors |  |  | Nurses |  |  | Leaflets |  |
| Friends |  |  | Neighbors |  |  | Others |  |

**Section C: Perception of falls and fractures.**

25.

| Statement | Strongly agree | Agree | Neither Agree or Disagree | Disagree | Strongly disagree |
| --- | --- | --- | --- | --- | --- |
| (a) Older people fall and there is nothing that can be done to prevent falls. |  |  |  |  |  |
| (b) It isn’t possible for me to fall down and get injured or fractured. |  |  |  |  |  |

| (c) I don’t worry about falling down and getting injured. |  |  |  |  |  |
| --- | --- | --- | --- | --- | --- |
| (d) The safety of my house is very good. |  |  |  |  |  |
| (e) I’m weak and need to do fall intervention activities. |  |  |  |  |  |
| (f) Intervention given after the first fall can prevent recurrent falls. |  |  |  |  |  |
| (g) Carrying out knowledge training program in fall induced injury in the community is a great necessity. |  |  |  |  |  |
| (h) Paying attention to correct my medical conditions is very important. |  |  |  |  |  |

**Section D: Attitude towards falls and fracture**

26.

| **Statement** | **Strongly**  **agree** | **Agree** | **Neither Agree nor Disagree** | **Disagree** | **Strongly disagree** |
| --- | --- | --- | --- | --- | --- |
| (a) I adjust my bed according to my convenience to prevent from falling |  |  |  |  |  |
| (b) I seek information related to falls and fracture |  |  |  |  |  |
| (c) I inform my caregivers if I experience any falls incidents |  |  |  |  |  |
| (d) When I do fall, I seek help |  |  |  |  |  |
| (e) I make sure the pathways are free from clutter |  |  |  |  |  |
| (f) If I experience any fall-related injury, I contact the emergency services for further medical attention |  |  |  |  |  |
| (g) I ensure the staircase and walkways are well lit |  |  |  |  |  |
| (h) I stop taking my medications when experiencing dizziness or giddiness |  |  |  |  |  |

**“Thank you for your participation in this study”**
