# Supplementary material for: Enhancing knowledge, attitude, and perceptions towards fall prevention among older adults: a pharmacist-led intervention in a primary healthcare clinic, Gemas, Malaysia
Source: BMC Geriatr. 2024 Apr 2;24:309. doi: 10.1186/s12877-024-04930-5 (PMC10988811; doi:10.1186/s12877-024-04930-5)
Supplement: Supplementary file 2 — Supplementary Material 2 [file 12877_2024_4930_MOESM2_ESM.docx]

**Supplementary Table 2** Medical conditions among the respondents (n=310)

| **Diseases** | **Frequency** | **Percentage (%)** |
| --- | --- | --- |
| Type 2 diabetes mellitus (T2DM) + hypertension + dyslipidaemia | 139 | 44.84 |
| Hypertension + dyslipidaemia | 87 | 28.06 |
| T2DM + hypertension | 15 | 4.84 |
| T2DM + dyslipidaemia | 11 | 3.55 |
| T2DM + hypertension + dyslipidaemia + myocardial infarction (MI) | 8 | 2.58 |
| Dyslipidaemia | 7 | 2.26 |
| COPD + hypertension + dyslipidaemia | 5 | 1.61 |
| Hypertension | 5 | 1.61 |
| T2DM + hypertension + dyslipidaemia + asthma | 4 | 1.29 |
| Hypertension + dyslipidaemia + MI | 3 | 0.97 |
| Hypertension + dyslipidaemia + gout | 2 | 0.65 |
| Hypertension + dyslipidaemia + asthma | 2 | 0.65 |
| T2DM+ dyslipidaemia + hypertension + gout | 2 | 0.65 |
| T2DM+ hypertension + epilepsy | 2 | 0.65 |
| MI + hypertension | 2 | 0.65 |
| T2DM + hypertension + dyslipidaemia + COPD | 2 | 0.65 |
| COPD | 2 | 0.65 |
| Chronic kidney disease (CKD) + hypertension + dyslipidaemia + gout | 1 | 0.32 |
| COPD + hypertension | 1 | 0.32 |
| T2DM + hypertension + dyslipidaemia + depression | 1 | 0.32 |
| Stroke + hypertension + dyslipidaemia | 1 | 0.32 |
| Atrial fibrillation + hypertension + dyslipidaemia | 1 | 0.32 |
| T2DM + hypertension + dyslipidaemia + hyperthyroidism | 1 | 0.32 |
| Dyslipidaemia + MI | 1 | 0.32 |
| T2DM + hypertension + dyslipidaemia + epilepsy | 1 | 0.32 |
| T2DM + hypertension + dyslipidaemia + hypothyroidism | 1 | 0.32 |
| Myocardial infarction | 1 | 0.32 |
| Hypertension + epilepsy | 1 | 0.32 |
| COPD + dyslipidaemia | 1 | 0.32 |
| Total | 310 | 100 |

**Supplementary Table 3** FRIDs prescribed to the respondents (n=310)

| **Drug classes & FRIDs** | **Frequency** | **Percentage (%)** |
| --- | --- | --- |
| **Cardiovascular** | **23** | **71.88** |
| Simvastatin | 259 | 83.55 |
| Amlodipine | 232 | 74.84 |
| Perindopril | 163 | 52.58 |
| Aspirin | 69 | 22.26 |
| Hydrochlorothiazide | 58 | 18.71 |
| Losartan | 32 | 10.32 |
| Prazosin | 30 | 9.68 |
| Atenolol | 28 | 9.03 |
| Bisoprolol | 24 | 7.74 |
| Atorvastatin | 15 | 4.84 |
| Frusemide | 11 | 3.55 |
| Glyceryl trinitrate (sublingual) | 8 | 2.58 |
| Telmisartan | 7 | 2.26 |
| Metoprolol | 7 | 2.26 |
| Isosorbide dinitrate | 3 | 0.97 |
| Spironolactone | 3 | 0.97 |
| Trimetazidine | 2 | 0.65 |
| Felodipine | 1 | 0.32 |
| Enalapril | 1 | 0.32 |
| Propranolol | 1 | 0.32 |
| Fenofibrate | 1 | 0.32 |
| Gemfibrozil | 1 | 0.32 |
| Warfarin | 1 | 0.32 |
| **Endocrine** | **5** | **15.62** |
| Metformin | 171 | 55.16 |
| Gliclazide | 67 | 21.61 |
| Basal bolus insulin | 35 | 11.29 |
| Premixed insulin | 15 | 4.84 |
| Basal insulin | 8 | 2.58 |
| **Central nervous system** | **4** | **12.50** |
| Sodium valproate | 2 | 0.65 |
| Amitriptyline | 1 | 0.32 |
| Carbamazepine | 1 | 0.32 |
| Phenytoin | 1 | 0.32 |

|  | **Pre-intervention (n=310)** | | | **Post-intervention (n=310)** | | |  |
| --- | --- | --- | --- | --- | --- | --- | --- |
| **Statements** | **Yes**  **n(%)** | **No**  **n(%)** | **Don’t know**  **n(%)** | **Yes**  **n(%)** | **No**  **n(%)** | **Don’t know**  **n(%)** | **p-value** |
| Falls and related fractures are the leading causes of hospital admission among elderly people | 232 (74.84) | 31 (10.0) | 47 (15.16) | 257 (82.91) | 25 (8.06) | 28 (9.03) | 0.00 |
| **Falls prevention knowledge** |  |  |  |  |  |  |  |
| a) Proper nutrition is very important to maintain your bone and muscle health which helps to protect you from falls. | 283 (91.29) | 6 (1.94) | 21 (6.77) | 296 (95.48) | 4 (1.29) | 10 (3.23) | 0.00 |
| b) Regular exercise and active lifestyle help to reduce the chance of falling | 281 (90.65) | 4 (1.29) | 25 (8.06) | 293 (94.51) | 3 (0.97) | 14 (4.52) | 0.00 |
| (c) When taking medication, if you follow proper medical advice, you can minimize the chances of falls due to side effects of drugs. | 294 (94.84) | 8 (2.58) | 8 (2.58) | 306 (98.70) | 2 (0.65) | 2 (0.65) | 0.00 |
| (d) Under favourable circumstances with good lighting, clean and clutter free floor, the risk of falls can be lowered. | 289 (93.23) | 8 (2.58) | 13 (4.19) | 297 (95.81) | 5 (1.61) | 8 (2.58) | 0.00 |

**Supplementary Table 4** Fall prevention knowledge among the respondents (n=310)

* Pearson correlation values between 0.5 and 1.0 shows a strong positive correlation.

**Supplementary Table 5** Risk factors for falls among the elderly (n=310)

| **Items /Questions** | **Pre-intervention (n=310)** | | **Post-intervention (n=310)** | | **r-value** | **p-value** |
| --- | --- | --- | --- | --- | --- | --- |
|  | **Yes**  **N (%)** | **No**  **N (%)** | **Yes**  **N (%)** | **No**  **N (%)** |  |  |
| **According to your knowledge, which of the following are risk factors for falls among people in your age?** |  |  |  |  |  |  |
| Biological factors such as age, gender, visual impairment, chronic diseases | 241 (77.74) | 69 (22.26) | 296 (95.48) | 14 (4.52) | 0.37* | 0.00* |
| Unsafe environment | 237 (76.45) | 73 (23.55) | 303 (97.74) | 7 (2.26) | 0.27* | 0.00* |
| Behavioural factors such as lack of physical activity, alcoholism | 144 (46.45) | 166 (53.55) | 241 (77.74) | 69 (22.26) | 0.49* | 0.00* |
| Socioeconomic factors (low income, difficulties to accessing health facilities) | 74 (23.87) | 236 (76.13) | 134 (43.23) | 176 (56.77) | 0.64* | 0.00* |
| Medication/Medicines | 82 (26.45) | 228 (73.55) | 216 (69.68) | 94 (30.32) | 0.39* | 0.00* |
| None of the above | 38 (12.26) | 0 (0) | 1 (0.32) | 0 (0) |  |  |
| **Medical conditions that may lead to a person falling include,** |  |  |  |  |  |  |
| Parkinson’s disease | 10 (3.23) | 300 (96.77) | 37 (11.94) | 273 (88.06) | 0.44* | 0.00* |
| Hypertension | 236 (76.13) | 74 (23.87) | 263 (84.84) | 47 (15.16) | 0.73* | 0.00* |
| Diabetes | 81 (26.13) | 229 (73.87) | 116 (37.42) | 194 (62.58) | 0.75* | 0.00* |
| Bone disorders | 63 (20.32) | 247 (79.68) | 100 (32.26) | 210 (67.74) | 0.73* | 0.00* |
| None of the above | 55 (17.74) | 0 (0) | 25 (8.06) | 0 (0) |  |  |
| **A fall may result in,** |  |  |  |  |  |  |
| Reduced mobility | 280 (90.32) | 30 (9.68) | 293 (96.52) | 17 (5.48) | 0.74* | 0.00* |
| Restriction of activities | 281 (90.65) | 29 (9.35) | 288 (92.90) | 22 (7.10) | 0.86* | 0.01* |
| Social isolation | 139 (44.84) | 171 (55.16) | 138 (44.52) | 172 (55.48) | 0.99* | 0.32 |
| None of the above | 24 (7.74) | 0 (0) | 13 (4.19) | 0 (0) | - |  |

* Pearson correlation values between 0.5 and 1.0 shows a strong positive correlation, 0.3 and 0.5 shows a medium correlation and 0.1-0.3 shows less correlation. p-value < 0.05 shows a significant difference

**Supplementary Table 6** The attitudes of the respondents towards falls and fractures (n=310)

| Statement | **Pre-intervention (n=310)** | | | | | | **Post-intervention (n=310)** | | | | | | r-value | p-value |
| --- | --- | --- | --- | --- | --- | --- | --- | --- | --- | --- | --- | --- | --- | --- |
|  | Strongly  Agree  n(%)  5 | Agree  n(%)  4 | Neither Agree nor Disagree  n(%)  3 | Disagree  n(%)  2 | Strongly disagree  n(%)  1 | Mean ±SD  n(%) | Strongly  Agree  n(%)  5 | Agree  n(%)  4 | Neither Agree nor Disagree  n(%)  3 | Disagree  n(%)  2 | Strongly disagree  n(%)  1 | Mean ±SD  n(%) |  |  |
| (a) I adjust my bed according to my convenience to prevent from falling | 0 (0) | 6 (1.93) | 1 (0.32) | 303 (97.74) | 0 (0) | 2.04 ± 0.08 | 0 (0) | 5 (1.61) | 1 (0.32) | 304 (98.07) | 0 (0) | 2.04 ± 0.07 | 0.77* | 0.56 |
| (b) I will seek information related to falls and fracture | 0 (0) | 16 (5.16) | 3 (0.97) | 290 (93.55) | 1 (0.32) | 2.11 ± 0.21 | 0 (0) | 58 (18.71) | 3 (0.97) | 249 (80.32) | 0 (0) | 2.38 ± 0.62 | 0.45* | 0.00* |
| (c) I inform my caregivers if I experience any falls incidents | 57 (18.39) | 248 (80.00) | 2 (0.64) | 3 (0.97) | 0 (0) | 4.16 ± 0.31 | 57 (18.39) | 241 (77.74) | 2 (0.64) | 10 (3.23) | 0 (0) | 4.11 ± 0.33 | 0.37* | 0.03* |
| (d) When I do fall, I seek help | 40 (12.90) | 266 (85.81) | 1 (0.32) | 3 (0.97) | 0 (0) | 4.11 ± 0.23 | 40 (12.90) | 268 (86.45) | 1 (0.32) | 1 (0.32) | 0 (0) | 4.12 ± 0.23 | 0.70* | 0.16 |
| (e) I make sure the pathways are free from clutter | 10 (3.23) | 293 (94.52) | 5 (1.61) | 2 (0.64) | 0 (0) | 4.00 ± 0.66 | 10 (3.23) | 297 (95.81) | 3 (0.97) | 0 (0) | 0 (0) | 4.02 ± 0.06 | *0.65 | 0.06 |
| (f) If I experience any fall-related injury, I contact the emergency services for further medical attention | 32 (10.32) | 273  (88.06) | 3 (0.97) | 2 (0.65) | 0 (0) | 4.08 ± 0.19 | 32 (10.32) | 277 (89.35) | 1 (0.32) | 0 (0) | 0 (0) | 4.10 ± 0.19 | 0.44* | 0.06 |
| (g) I ensure the staircase and walkways are well lit | 4 (1.29) | 289 (93.22) | 1 (0.32) | 16 (5.16) | 0 (0) | 3.91 ± 0.02 | 5 (1.61) | 302 (97.42) | 1 (0.32) | 2 (0.65) | 0 (0) | 4.00 ± 0.03 | 0.41* | 0.00* |
| (h) I stop taking my medications when experiencing dizziness or giddiness | 11 (3.55) | 221 (71.29) | 29 (9.35) | 48 (15.48) | 1 (0.32) | 3.62 ± 0.63 | 11 (3.55) | 278 (89.68) | 10 (3.23) | 11 (3.55) | 0 (0) | 3.93 ± 0.19 | 0.46* | 0.00* |

* Pearson correlation values between 0.5 and 1.0 shows a strong positive correlation; 0.3 and 0.5 shows a medium correlation.

*p-value <0.05 considered as statistically significant.

**Supplementary Table 7** Perception of the respondents on falls and fractures (n=310)

| Statement | **Pre-intervention (n=310)** | | | | | | **Post-intervention (n=310)** | | | | | | r-value | p-value |
| --- | --- | --- | --- | --- | --- | --- | --- | --- | --- | --- | --- | --- | --- | --- |
|  | Strongly Agree  n(%) | Agree  n(%) | Neither Agree nor Disagree  n(%) | Disagree  n(%) | Strongly disagree  n(%) | Mean ±SD  n(%) | Strongly  Agree  n(%) | Agree  n(%) | Neither Agree nor Disagree  n(%) | Disagree  n(%) | Strongly disagree  n(%) | Mean ±SD  n(%) |  |  |
| (a) Older people fall and there is nothing that can be done to prevent falls. | 50 (16.13) | 70 (22.58) | 24 (7.74) | 161 (51.94) | 5 (1.61) | 2.99 ± 1.10 | 24 (7.74) | 49 (15.81) | 24 (7.74) | 208 (67.10) | 5 (1.61) | 2.61 ±  0.87 | 0.72* | 0.00* |
| (b) It isn’t possible for me to fall down and get injured or fractured. | 0 (0) | 5 (1.61) | 5 (1.61) | 284 (91.62) | 16 (5.16) | 3.98 ± 0.10 | 0 (0) | 4 (1.29) | 4 (1.29) | 286 (92.26) | 16 (5.16) | 1.99 ± 0.10 | 0.89* | 0.18 |
| (c) I don’t worry about falling down and getting injured. | 5 (1.61) | 52 (16.78) | 7 (2.26) | 172 (55.48) | 74 (23.87) | 2.17 ± 0.74 | 5 (1.61) | 52 (16.77) | 6 (1.94) | 173 (55.81) | 74 (23.87) | 2.16 ± 0.74 | 0.99* | 0.66 |
| (d) The safety of my house is very good. | 0 (0) | 297 (95.80) | 2 (0.65) | 11 (3.55) | 0 (0) | 3.92 ± 0.15 | 0 (0) | 297 (95.80) | 2 (0.65) | 11 (3.55) | 0 (0) | 3.92 ± 0.15 | 0.92* | 1.00 |
| (e) I’m weak and need to do fall intervention activities. | 6 (1.94) | 147 (47.42) | 50 (15.48) | 107 (34.52) | 0 (0) | 3.17 ± 0.86 | 6 (1.94) | 157 (50.65) | 43 (13.87) | 104 (33.55) | 0 (0) | 3.21 ± 0.87 | *0.94 | 0.02* |
| (f) Intervention given after the first fall can prevent recurrent falls. | 0 (0) | 180  (58.06) | 115 (37.10) | 15 (4.84) | 0 (0) | 3.53 ± 0.54 | 0 (0) | 219 (70.65) | 79 (25.48) | 12 (3.87) | 0 (0) | 3.67 ± 0.47 | 0.76* | 0.00* |
| (g) Carrying out a knowledge training program in fall-induced injury in the community is a great necessity. | 3 (0.97) | 203 (65.48) | 100 (32.26) | 4 (1.29) | 0 (0) | 3.67 ± 0.46 | 3 (0.97) | 227 (73.22) | 78 (25.16) | 2 (0.65) | 0 (0) | 3.75 ± 0.40 | *0.83 | 0.00* |
| (h) Paying attention to correct my medical conditions is very important. | 16 (5.16) | 289 (93.23) | 5 (1.61) | 0 (0) | 0 (0) | 4.04 ± 0.10 | 16 (5.16) | 290 (93.55) | 4 (1.29) | 0 (0) | 0 (0) | 4.04 ± 0.10 | *0.89 | 0.32 |

* Pearson correlation values between 0.5 and 1.0 shows a strong positive correlation. p-value < 0.05 shows a significant difference

**Supplementary Table 8** Effectiveness of educational intervention based on sex and educational level of the respondents (n=310)

|  | **Knowledge** | | | **Perception** | | | **Attitude** | | |
| --- | --- | --- | --- | --- | --- | --- | --- | --- | --- |
|  | Pre | Post | p-value | Pre | Post | p-value | Pre | Post | p-value |
| **Sex** | | | | | | | | | |
| Male (n=141) | 8520 | 10045 | 0.00* | 4050 | 4151 | 0.00* | 3934 | 4044 | 0.00* |
| Female (n=169) | 10095 | 12455 | 0.00* | 4989 | 5092 | 0.00* | 4755 | 4859 | 0.00* |
| Total | 18615 | 22500 | - | 9039 | 9243 | - | 8689 | 8903 | - |
| **Education level** | | | | | | | | | |
| No formal education (n=12) | 610 | 710 | 0.00* | 347 | 349 | 0.01* | 336 | 341 | 0.01* |
| Primary education (n=229) | 13295 | 16365 | 0.00* | 6650 | 6829 | 0.00* | 6413 | 6580 | 0.00* |
| Secondary education (n=69) | 4665 | 5425 | 0.00* | 2042 | 2065 | 0.00* | 1940 | 1982 | 0.00* |
| Total | 18615 | 22500 | - | 9039 | 9243 | - | 8689 | 8903 | - |

*p-value <0.05 considered as statistically significant; Pre: Pre-intervention; Post: Post-intervention.
